# Experimental Analyses Emphasize the Stability of the Meisenheimer Complex in a S_N_Ar Reaction Toward Trends in Reaction Pathways

**DOI:** 10.3389/fchem.2020.00583

**Published:** 2020-07-10

**Authors:** Paola R. Campodónico, Belén Olivares, Ricardo A. Tapia

**Affiliations:** ^1^Centro de Química Médica, Facultad de Medicina, Clínica Alemana Universidad del Desarrollo, Santiago, Chile; ^2^Facultad de Química y Farmacia, Pontificia Universidad Católica de Chile, Santiago, Chile

**Keywords:** S_N_Ar reaction, reaction mechanism, Brönsted-type plots, border mechanisms, Meisenheimer complex

## Abstract

The mechanism of S_N_Ar reactions between 2-chloro-5-nitropyrimidine with primary and secondary alicyclic amines, respectively, have been studied by kinetic measurements. The kinetic data obtained in aqueous media opens a controversial discussion based on Brönsted-type plots analysis. The first approach based on the kinetic data reveals a non-catalyzed pathway. Then, the subtlety of the mathematical treatment of the kinetic data is discussed over a concerted or stepwise mechanism, respectively.

## Introduction

The nucleophilic aromatic substitution (S_N_Ar) reactions have been the object of several studies, especially in solvation analysis and solvent effects studies in water, conventional organic solvents (COS), ionic liquids (ILs), and mixtures of them (Nudelman et al., 1987; Newington et al., 2007; D'Anna et al., 2010; Park and Lee, 2010; Alarcón-Espósito et al., 2015, 2016, 2017; Marullo et al., 2016; Sánchez et al., 2018a,b). However, only in the last time these reactions have been investigated systematically in order to acquire knowledge about the rate determining step (RDS) on the reaction mechanism based on Brönsted type-plots (Um et al., 2007; Ormazábal-Toledo et al., 2013a,b; Gallardo-Fuentes et al., 2014; Gazitúa et al., 2014). This free energy relationship correlates the logarithm of the nucleophilic rate coefficients (*k*_*N*_) and the *pK*_*a*_ values of the nucleophiles from Brönsted Equation:

(1) $$\begin{array}{r} logk_{N}=\beta_{\text{nuc}}\;pK_{a}+log\;log\;G \end{array}$$

where *G* is a constant that depends of the solvent and temperature and β_*nuc*_ corresponds to the development of charge between the reaction sites of the nucleophile/electrophile pair, respectively, along to the potential energy surface (PES) (Brönsted and Pedersen, 1924). Therefore, β_*nuc*_ gives information about the transition state (TS) structure related to the RDS on the reaction mechanism (Buncel et al., 1993).

**Scheme 1:**
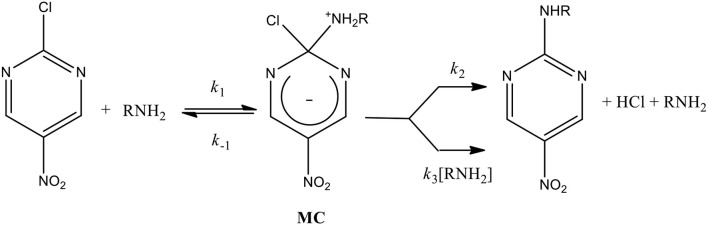
General reaction mechanism for a S_N_Ar reaction between 2-chloro-5-nitropyrimidine as substrate with primary amines as nucleophiles (general chemical structure).

The established mechanism for S_N_Ar reactions occurs in activated aromatic substrates with strong electron withdrawing groups (EWG) containing a good leaving group (LG) through an addition-elimination process (Crampton et al., 2004; Um et al., 2007; Ormazábal-Toledo et al., 2013a,b; Terrier, 2013; Gallardo-Fuentes et al., 2014; Gazitúa et al., 2014; Alarcón-Espósito et al., 2015, 2016, 2017; Mortier, 2015; Sánchez et al., 2018a,b). The first step of this stepwise mechanism is the nucleophilic attack to the substrate (*k*_1_ channel in Scheme 1) leading to the formation of an anionic σ-adduct named Meisenheimer complex (MC). Subsequent to the MC development,

two processes for its decomposition have been postulated: (i) expulsion of the LG followed by fast proton loss to give the reaction product (*k*_2_ channel in Scheme 1) and (ii) the base-catalyzed deprotonation of the zwitterionic complex that loss the LG to give the reaction product (*k*_3_ channel in Scheme 1) (Crampton et al., 2004; Um et al., 2007; Ormazábal-Toledo et al., 2013a,b; Terrier, 2013; Gallardo-Fuentes et al., 2014; Gazitúa et al., 2014; Alarcón-Espósito et al., 2015, 2016, 2017; Mortier, 2015; Sánchez et al., 2018a,b) Scheme 1 shows the general reaction mechanism for a S_N_Ar considering the reaction of this study and the decomposition channel cited above (*k*_2_ and *k*_3_, respectively) (Bunnett and Zahler, 1951; Bunnett and Cartano, 1981; Bunnett et al., 1981) See details in Scheme 1 and Results and Discussions.

Nowadays, researchers have opened the discussion over concerted vs. stepwise mechanisms on S_N_Ar reactions. For instance, Um et al. postulated a concerted route based on the evidence of a cyclic TS structure for the reaction between 1-(Y-substituted-phenoxy)-2,4-dinitrobenzenes with cyclic secondary amines in acetonitrile (Um et al., 2014). Recently, Jacobsen et al. provides experimental and computational evidences that S_N_Ar reactions proceed through concerted mechanism (Kwan et al., 2018) Ritter et al. has proposed concerted S_N_Ar mechanism based on theoretical analysis validated by experimental studies on the deoxyfluorination reaction of phenols (Neumann et al., 2016; Neumann and Ritter, 2017).

**Scheme 2:**
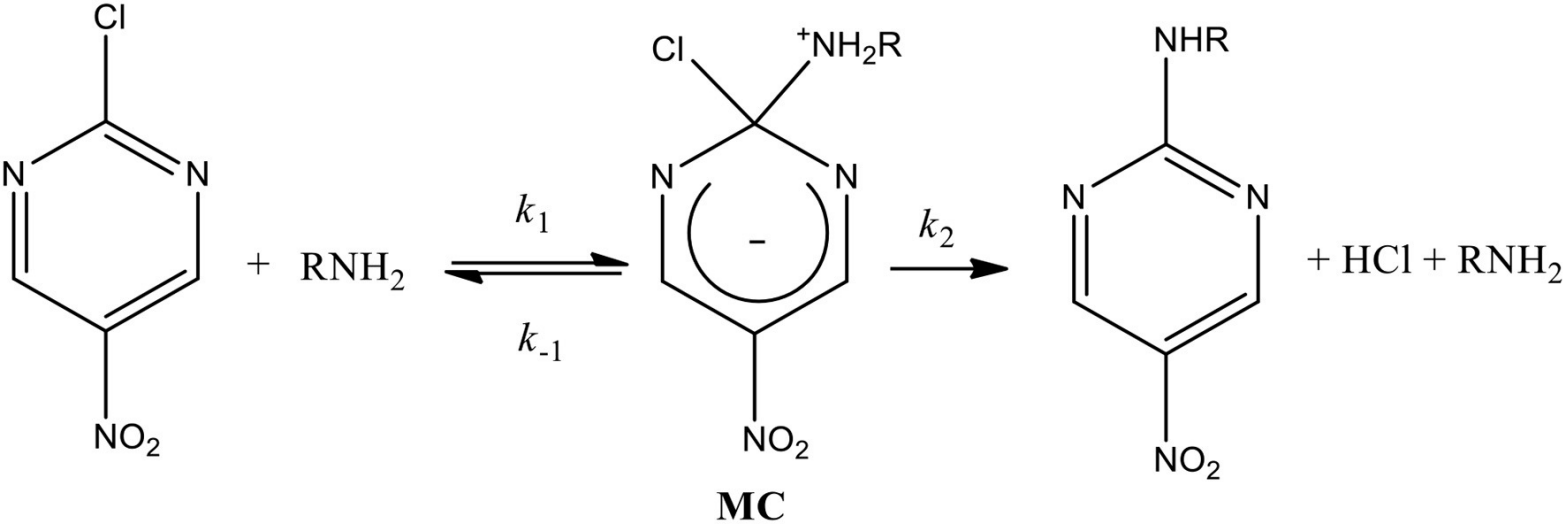
General reaction mechanism for a S_N_Ar reaction between 2-chloro-5-nitropyrimidine as substrate with primary amines as nucleophiles. It excludes the general-base catalyzed mechanism (*k*_3_ *in Scheme* 1).

In S_N_Ar reactions proceeding through a stepwise mechanism that discard the general-base catalyzed mechanism (*k*_3_ *in* *Scheme* 1), Crampton et al. obtained β_*nuc*_ values close to 0.5 which were attributed to the MC formation (nucleophilic attack) as RDS on the reaction mechanism (see Scheme 1) (Crampton et al., 2004, 2006; Um et al., 2007; Stenlid and Brinck, 2017). Up to date there are no reports that can establish a range for β_*nuc*_ values that could separate the nucleophilic attack and the LG departure steps on the reaction mechanism for S_N_Ar processes indicating a non-catalyzed pathway. For instance, β_*nuc*_ values were reported for the S_N_Ar reaction between 2,4-dinitrophenylsulfonylchloride with secondary alicyclic amines in aqueous media (Gazitúa et al., 2014), where the LG departure is the RDS for a non-catalyzed pathway (*k*_2_ channel in Scheme 1). This result was obtained using the β_*nuc*_ values proposed by Jencks in nucleophilic substitution reactions of carbonates with amine series, attributing β_*nuc*_ values of 1.0 for the LG departure and 0.3 for the nucleophilic attack, respectively, obtained from linear Brönsted-type plots (Jencks and Gilchrist, 1968) These analysis were performed considering that the reactions proceeded through: (i) a MC intermediate (stepwise mechanism) and (ii) non-catalyzed pathway, both in agreement to Scheme 2. However, the stability of the MC opens the possibility toward a concerted pathway (without MC) instead of a stepwise mechanism (Gallardo-Fuentes and Ormazábal-Toledo, 2019).

On the other hand, linear Brönsted-type plots for nucleophilic substitution have been associated with concerted mechanisms, in which the nucleophilic attack at the electrophilic center occurs concertedly with the LG departure within a single step pathway (Perrin, 2000). A range of slope values have been reported by Castro et al. from 0.40 to 0.60 for aminolysis of carboxylic esters derivatives (Castro, 1999). Note that, this range of β_*nuc*_ values assigned to concerted mechanisms for nucleophilic substitution is similar to β_*nuc*_ values associated to the nucleophilic attack in S_N_Ar reactions.

Plotting log *k*_*N*_ and *pK*_*a*_ values in the traditional way affording curved Brönsted type-plots have been reported. The later, can be associated to a stepwise mechanism with a change in the TS structure associated to the RDS. Statistically downward curvatures for corrected Brönsted-type plots defined β_*nuc*_ values associated to the LG departure in the range between 0.8–1.1 and 0.1–0.3 for the nucleophilic attack, respectively (Jencks and Gilchrist, 1968; Williams, 1989). Subsequently, the above range was also obtained by Castro et al. in the reaction of carbonate and thiocarbonate derivatives with *N*-nucleophiles series (Jencks and Gilchrist, 1968; Perrin, 2000). However, other analysis associated to curved Brönsted-type plots postulate a concerted pathway. In this case, the reported mechanism is attributable to strong EWG in the electrophile (substrate) that could destabilize the hypothetical intermediate (in a hypothetical stepwise mechanism, see Scheme 2) (Castro et al., 2001, 2002a; Knipe, 2019). Note that, the difference between nucleophilic substitution and S_N_Ar reactions is the type of intermediate given by the nature of the reacting pair. For nucleophilic substitution is postulated a zwitterionic tetrahedral intermediate (T^±^) (Satterthwait and Jencks, 1974; Castro et al., 2002a), while in a S_N_Ar reaction is suggested an anionic σ-adduct (MC in Schemes 1, 2, respectively).

Jacobsen et al. summarize the mechanistic trends in S_N_Ar reactions centered on the chemical structures of the substrates, specifically groups or atoms attached to the permanent group (PG) and the nature of the LG as shown below (Kwan et al., 2018).

**Table:** 

**PG**	**LG**	**Suggested Mechanism**
Strong EWG	Poor	Stepwise
Heterocycles that contain nitrogen atoms	Good	Concerted
Strong EWG	Good	Borderline

Note that, this description excludes the nucleophile nature. Nevertheless, it is known that the nature of the reacting pair (solute) plays a key role on the reactivity added to the hydrogen bond (HB) effect involved in solute-solvent interactions over the stabilization of species along the PES for S_N_Ar reactions (Bernasconi and De Rossi, 1976; Newington et al., 2007; Ormazábal-Toledo et al., 2013a,b; Gallardo-Fuentes et al., 2014; Sánchez et al., 2018a,b). Furthermore, it is known that secondary alicyclic amines (SAA) are better nucleophiles than primary amines (PA) (Ormazábal-Toledo et al., 2013a,b). On the other hand, the substrate or electrophile is highly reactive, because it contains nitrogen atoms in its chemical structure added to an EWG group and chlorine is considered a good LG. Then, applying the table that summarize the mechanistic trends based on the nature of the reacting pair, the possibility for a reaction between SAA and 2-chloro-5-nitropyrimidine suggests a concerted pathway. However, depending of the nature of the LG, two routes could be possible: (i) poor LG, the LG departure step will be the RDS (stepwise mechanism) and (ii) good LG, the nucleophilic attack could be occurring at the same time to the leaving group departure or the nucleophilic attack will be the rate determining step. Therefore, the meaning of a borderline mechanism will be the uncertainty of which reaction channel will follow a solute.

*As a result, the number of factors controlling reactivity in S*_*N*_*Ar reactions is large, and could produce effects over the reaction mechanism given by the stabilization of the MC toward stepwise (stwS*_*N*_*Ar) or concerted mechanisms (cS*_*N*_*Ar)*.

In this context, we have recently studied the reactivity of 2-chloro-5-nitropyrimidine with benzohydrazides, establishing that the HB between the reacting pair activate the pyrimidine moiety and increases the nucleophilicity of the nitrogen atom of the benzohydrazide fragment (Campodónico et al., 2010; Gallardo-Fuentes et al., 2014). Note that, in this case the nucleophile series are not very reactive, promoting a *stw*S_N_Ar mechanism, where the nucleophilic attack is the RDS of the reaction mechanism (see Scheme 1). Other studies for nitrogen heterocyclic compounds have shown a borderline mechanism, such as the reaction between atrazine and bio-thiols (Calfumán et al., 2017). On the other hand, the reaction between 4-chloroquinazoline with aniline suggested a *stw*S_N_Ar route being the first step (nucleophilic attack) the RDS of the reaction mechanism (Sánchez et al., 2018b). These previous kinetic studies and others based on heterocyclic substrates that contain nitrogen atoms in its chemical structures opened an interesting discussion over mechanistic features of S_N_Ar reactions (Taylor and Thompson, 1961; Cherkasov et al., 1982; Cullum et al., 1995, 1996), especially with ambident substrates, which would be showing more than one site toward the nucleophilic attack and another alternative mechanism (Guo and Mayr, 2014; Gabsi et al., 2018).

**Scheme 3:**
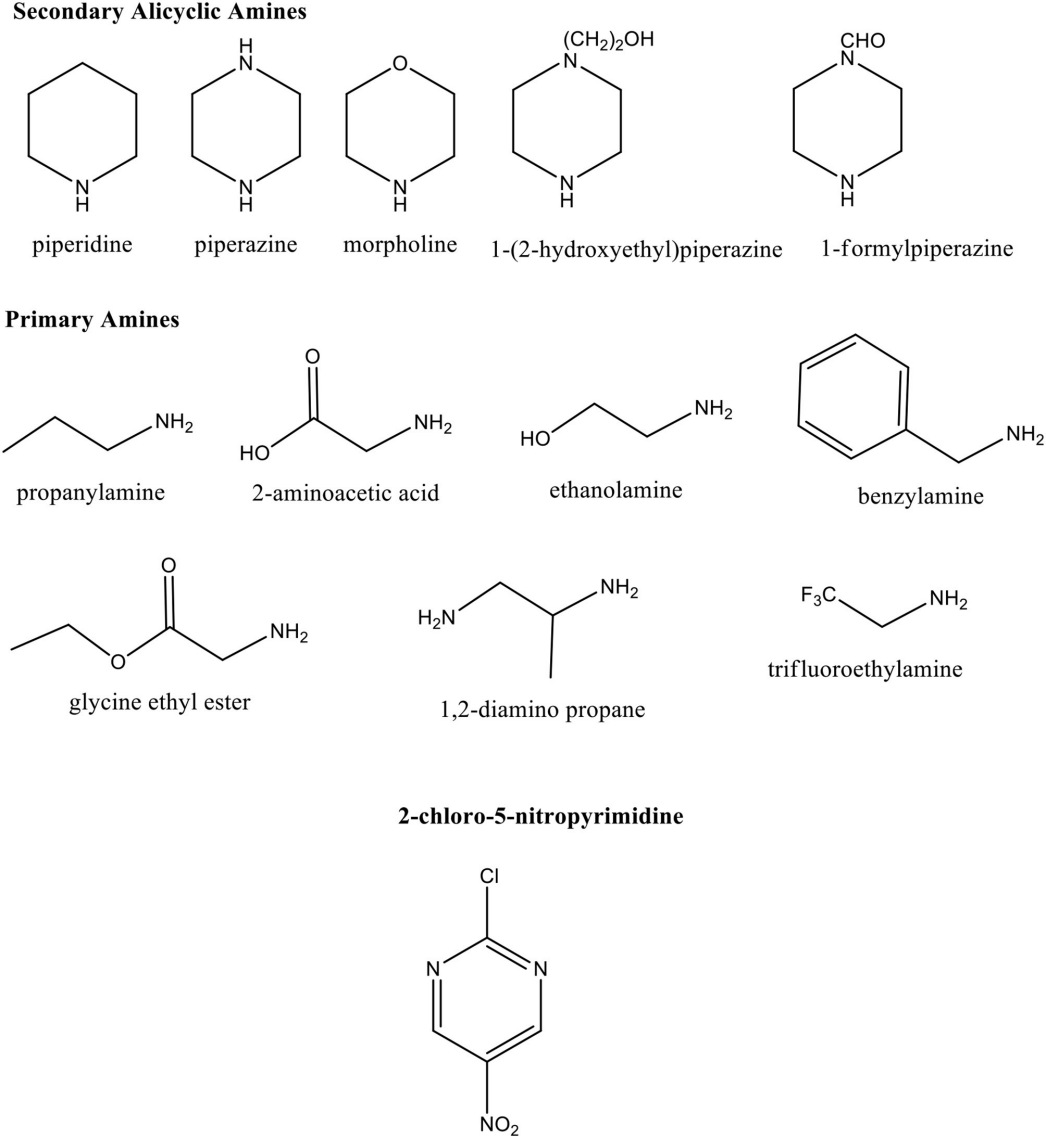
Chemical structures of secondary alicyclic and primary amines, respectively and the substrate 2-chloro-5-nitropyrimidine.

In this work, we report a complete kinetic study to better understand the reaction mechanism which is operative between the substrate 2-chloro-5-nitropyrimidine with two series of N-nucleophiles: PA and SAA in aqueous media (see Scheme 3). Both series of nucleophiles proceed through of a S_N_Ar route for which reaction mechanisms were assigned. These results were discussed in terms of the comparison of Brönsted slope parameters (β_*nuc*_) given by the Brönsted type-plots analysis, as well as examination of chemical structures of the reacting pairs.

## Materials and Methods

### Reactants and Solvents

All the reagents used were the commercially available by Sigma-Aldrich and Merck. The certificate of analysis guarantees purity ≥ 99%.

### Kinetic Measurements

The kinetics of the studied reactions were carried out spectrophotometrically by means of a diode array spectrophotometer for slow reactions and a stopped-flow spectrophotometer for fast reactions equipped with a constant temperature circulating bath. In aqueous solution, the experimental conditions were 25.0 ± 0.1°C, ionic strength 0.2 M (KCl), at three different pH values maintained by partial protonation of the amines. All the reactions were studied under excess of the amine over the substrate and were started by injection of a substrate stock solution in acetonitrile (10 μL) into the amine solution (2.5 mL in the spectrophotometric cell). The initial substrate concentration was about 0.1 M. The pseudo first order constant (*k*_obs_) values were found for all reactions; which were determined by means of the spectrophotometer kinetic software for first order reactions at the wavelength corresponding at the kinetic products. Note that, in aqueous media each pH values correspond to: pH = *pK*_*a*_ and 0.3 units up and down in order to analyze the possibility of acid and/or basic catalysis by the reaction media. On the other hand, a Brönsted type-plot requires a broad range of *pK*_*a*_ values for the nucleophiles. For this reason, in this study is used a family of nucleophiles with similar chemical features, 7 SAA and 7 PA, respectively. Then, the relationships between *k*_*obs*_ vs. [Nucleophile] should be straight lines or straight lines with smooth deviations, which will discard a catalysis processes by the media. All the reactions were carried out under pseudo first-order conditions in which the amine concentrations were at least 10 times greater than the substrate concentration (Um et al., 2007, 2012).

### Product Analysis

In the studied reactions, the increase of a band centered in the range of 330–550 nm was observed; and it was attributed to the corresponding 5-nitro-(2-amine-1-yl) pyrimidine like reaction products for all amines studied.

### Synthesis of Products

#### 5-Nitro-*N*-Propylpyrimidin-2-Amine

To a mixture of 2-chloro-5-nitropyrimidine (40 mg, 0.25 mmol) in tetrahydrofuran (THF, 5 mL), containing triethylamine (25.3 mg, 0.25 mmol), was added dropwise a solution of piperidine (14.8 mg, 0.25 mmol) in THF (2.0 mL). The reaction mixture was stirred for 20 h at room temperature, the solvent was removed under vacuum and the residue was dissolved in ethyl acetate (15 mL). The organic layer was washed with 0.5 N HCl, water, brine and dried over Na_2_SO_4_. After evaporation of the solvent the crude product was purified by flash chromatography on silica gel (CH_2_Cl_2_–hexane 1:1) to give a yellow solid (35 mg, 77%), mp 117–118°C (Lit. Barlin and Young, 1972 116°C). IR (KBr) ν_max_ cm^−−*l*^ 3246, 1602, 1580, 1560, 1334, 1300. ^1^H-NMR (200 MHz, CDCl_3_) ð*: 1.01 (t, *J* = 7.2 Hz, 3H), 1.68 (sext, *J* = 7.2 Hz, 2H), 3.51 (q, *J* = 7.2 Hz, 2H), 6.09 (br s, 1H), 9.03 (s, 1H), 9.10 (s, 1H); ^13^C-NMR (50.4 MHz, CDCl_3_/DMSO-*d*_6_) ð*: 11.5, 22.3, 43.6, 133.6, 154.9, 155.2, 163.3.

#### 5-Nitro-2-(Piperidin-1-yl)Pyrimidine

Using the above procedure, from 2-chloro-5-nitropyrimidine (40 mg, 0.25 mmol) and piperidine (22 mg, 0.258 mmol), was obtained a yellow solid (27 mg, 52%), mp 154–156°C (Lit. Boarland and McOmie, 1951147–148°C). IR (KBr) ν_max_ cm^−−l^ 1602, 1580, 1332, 1303. ^1^H-NMR (200 MHz, CDCl_3_) ð*: 1.60–1.80 (m, 6 H), 3.95 (t, *J* = 5.0 Hz, 4 H), 8.90 (s, 2H); ^13^C-NMR (50.4 MHz, CDCl_3_) ð*: 23.6, 25.1 (2C), 44.8 (2C), 99.4, 154.1 (2C), 167.2. HRMS (ESI) calcd for C_9_H_12_N_4_O_2_[M +] 208.0960, found 208.0955.

## Results and Discussion

Under the experimental conditions used, the formation of only one product was spectrophotometrically observed for all the reactions studied. Therefore, the possibility of nucleophilic attack at the unsubstituted ring positions, substitution of hydrogen (Gabsi et al., 2018), was discarded (Um et al., 2007). This fact was confirmed by synthesis and study of the reaction products (see Experimental Section and Supplementary Material), discarding the possibility of nucleophilic attack at the unsubstituted positions on the aromatic ring (4 and 6, positions).

The kinetic study for the reaction of 2-chloro-5-nitropyrimidine with the whole set of amines considered in this study (see Scheme 3 and Table 1) was performed in aqueous solution at 25°C and ionic strength 0.2 M in KCl. The formation of colored amino-substituted nitropyrimidine compounds were monitored by UV–vis spectroscopy. In all runs, an excess of amines over the substrate were used in order to achieve pseudo-first-order kinetic conditions and the pseudo-first-order rate constant (*k*_*obs*_) was found for all the reactions. See more details in Figures S1–S14 and Tables S1–S42, respectively in Supplementary Material.

**Table 1:** Summary of nucleophiles and their statistically corrected *pK*_*a*_ values in water and the second-order rate constant (*k*_*N*_) for the nucleophile series with 2-chloro-5-nitropyrimidine.

**Name**	**pK_**a**_**	***k*_**N**_ (sM)^**−1**^**
**PRIMARY AMINES**		
Propylamine	11.14	6.93 ± 0.47
Glycine	10.24	3.16 ± 0.09
Ethanolamine	9.98	1.95 ± 0.04
Benzylamine	9.82	2.78 ± 0.06
Glycine ethyl ester	8.23	0.43 ± 0.01
1,2-Diamino propane	7.31	0.220 ± 0.007
Trifluoroethylamine	6.18	0.0260 ± 0.00005
**SECONDARY ALICYCLIC AMINES**		
Piperidine	11.54	100 ± 2.78
Piperidine*	11.54	52.39 ± 2.10
Piperazine	9.94	65.2 ± 2.50
1–(2–Hidroxyethyl) piperazine	9.38	50 ± 1.50
Morpholine	8.78	41.6 ± 0.95
1–Formylpiperazine	7.93	20 ± 0.52
Piperazinium anion	6.28	1.83 ± 0.052

* *Kinetic data for piperidine obtained using stop flow equipment*.

The kinetic analysis (Terrier, 2013; Mortier, 2015) shows that the *k*_*obs*_ for the studied reactions can be expressed as Equation (2), in which [*Nu*] represents the concentration of nucleophile and *k*_1_, *k*_2_ and *k*_3_ are the micro-constants associated to the reaction mechanism of a S_N_Ar reaction (see Scheme 1).

(2) $$\begin{array}{r} k_{obs}\;=\;\frac{k_{1}k_{2}\left[Nu\right]\;+\;k_{1}k_{3}{\left[Nu\right]}^{2}}{k_{-1}+\;k_{2}+\;k_{3}\left[Nu\right]} \end{array}$$

The values of *k*_*obs*_ for all the reactions are in accordance with Equation (3) where *k*_0_ and *k*_*N*_ are the rate coefficients for hydrolysis and aminolysis, respectively. Then, the *k*_*obs*_ values were obtained at different concentrations of the nucleophile in aqueous media. These results were plotted through of *k*_*obs*_ vs. [*Nu*] in order to obtain the *k*_*N*_ values from Equationn 3:

(3) $$\begin{array}{r} k_{obs}=\;k_{0}\;+\;k_{N}\left[Nu\right] \end{array}$$

These linear plots passed through the origin or close to it, suggesting that the contribution of hydroxide and/or water to *k*_*obs*_ values is negligible and the reactions do not follow a catalyzed route in agreement with Scheme 2 (*k*_−1_ + *k*_2_ ≫ *k*_3_[*Nu*] *in* *Scheme* 1) (see more details in SM). Then, *k*_*obs*_ is expressed by Equation 4:

(4) $$\begin{array}{r} k_{obs}\;=\;k_{N}\left[Nu\right],\;where\;k_{N}\;=\;\frac{k_{1}k_{2}}{\left(k_{-1}\;+\;k_{2}\right)} \end{array}$$

Table 1 summarizes the *pK*_*a*_ values in water and the rate coefficients values obtained for the amination of the pyrimidine derivative in aqueous media at 25°C and ionic strength 0.2 M in KCl. The *pK*_*a*_ and *k*_*N*_ values were statistically corrected using *p* (numbers of protons which can be deprotonated from the conjugate acid of the amine) and *q* (numbers of nucleophilic sites of the amine). The value accompanying *k*_*N*_ coefficients corresponds to the error associated to the slope to obtain these values (Bell, 1973).

**Figure 1:**
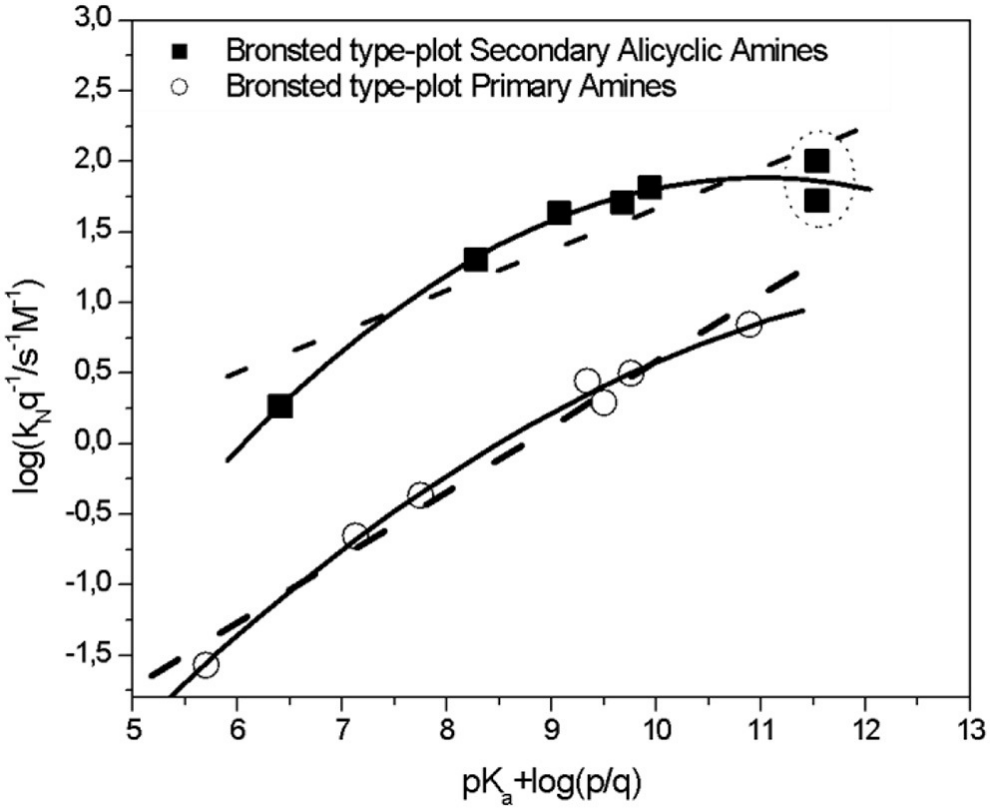
Brönsted-type plots (statistically corrected) obtained for the reactions of 2-choro-5-nitropyrimidine with Secondary Alicyclic Amines series (full squares, see Scheme 3 and Table 1) and Primary Amines series (empty circles, see Scheme 3 and Table 1), respectively in aqueous solution, at 25.0°C and ionic strength of 0.2 M in KCl. Data enclosed in dots correspond to piperidine. Continued and dotted lines correspond to the free energy relationships that correlates the logarithm of the nucleophilic rate coefficients (*k*_*N*_) and the *pK*_*a*_ values of the nucleophiles from Brönsted Equation. See below in the text the analysis of those trends.

Figure 1 shows the Brönsted-type plots for the studied reactions in agreement to Equation 1, where the *k*_*N*_ and *pK*_*a*_ values were statistically corrected by *p* and *q* parameters, respectively. The analysis of the statistically corrected Brönsted-type plots is based on the discussion of two trends (linear and curve) for each amine series (see Scheme 3) in order to elucidate the RDS on the reaction mechanisms if its channel follows a stepwise route or to get highlights about the meaning of curved Brönsted-type plots.

First part in Figure 1 (empty circles in bottom) corresponds to PA serie. Note for these amines, that the rate coefficients increase together with its *pK*_*a*_ values showing a weak curvature. However, it will perfectly be a linear trend (see dotted and continued lines in Figure 1). From this linear behavior, a β_*nuc*_ value of 0.46 ± 0.03 is obtained, which is comparable to those reported for the S_N_Ar reaction of 2,4-dinitrochlorobenzene with SAA and PA in water, suggesting that the RDS is the nucleophilic attack (Um et al., 2007, 2012) For this serie, with *N* = 7, the parameters for a straight line are: *R*^2^ = 0.976, SD = 0.141 and *p* < 0.0001, respectively. On the other hand, and maintaining N, the values for the curved plot (polynomial fit of 2nd order) are *R*^2^ = 0.994, SD = 0.077 and *p* < 0.0001, respectively.

Second part in Figure 1 (full squares in top) corresponds to SAA. Note that for these nucleophiles, the curvature is more stressed in comparison with PA, specifically toward the most basic amine (piperidine). See the comparison between continued and dotted lines. Therefore, for the complete series of these amines the strength trend is discarded (see Figure 1, dotted line). However, a rude first approximation for these SAA, will be eliminating the *k*_*N*_ value obtained for piperidine, obtaining a β_*nuc*_ value of 0. 44 ± 0.04. Note that, this value will be comparable with the β_*nuc*_ value obtained for PA suggesting the same reaction route. For this serie, with *N* = 8, the parameters for a straight line are: *R*^2^ = 0.791, SD = 0.271 and *p* = 0.003, respectively.

Continuing with the same section of the Figure 1, a new analysis considering all the kinetic data for the SAA shown a Brönsted-break plot (see continued line in Figure 1). Note that, all the kinetic data in this study were carried out spectrophotometrically by a diode array spectrophotometer. However, piperidine shows kinetic measurements extremely fast and high *k*_*obs*_ values (see Figure S8 and Tables S22–S24, respectively in SM). This fact was corrected using a stop flow equipment connected to the diode array spectrophotometer maintaining the same experimental conditions. The *k*_*obs*_ values are reported in Figure S14 and Tables S40–S42, respectively in SM and *k*_*N*_ value is shown in Table 1. Note that, this *k*_*N*_ value emphases the curvature on the Brönsted-type plot (see dotted circle in Figure 1 and data in Table 1). Maintaining *N* = 8, the parameter values for the curved plot (polynomial fit of 2nd order) are *R*^2^ = 0.977, SD = 0.093 and *p* < 0.0001, respectively.

Castro et al. reported a similar behavior in aminolysis of carbonate derivatives, suggesting a concerted mechanism (Castro et al., 2002a). The observed curvature on the Brönsted-type plots given by the reaction between piperidine and 2-choro-5-nitropyrimidine, will be attributed to the electron-withdrawing effect of the nitro group added to the high nucleophilic strength of piperidine. The synergy of both effects over the reaction would destabilize the MC intermediate in a hypothetical stepwise process promoting the concerted route. This fact added to the Jacobsen et al. analysis: heterocycles that contain nitrogen atoms plus good LG follow a cS_N_Ar route (Kwan et al., 2018).

Then, if a Brönsted-type plot is considered as a definitive proof to validate a concerted mechanism, it implies the prediction of the *pK*_*a*_ position at the break of the biphasic Brönsted type-plot ($pK_{a}^{0}$) for the hypothetical stepwise mechanism. This condition establish that this value should fall within the *pK*_*a*_ range of the amines employed (Chrystiuk and Williams, 1987; Williams, 1989; Castro et al., 2006). It is also important to obtain a large number of data, which cover a substantial *pK*_*a*_ range above and below the $pK_{a}^{0}$ value (Williams, 2007). Equation 5 is a semiempirical equation of 4 parameters based on the existence of an intermediate on the reaction mechanism (Castro et al., 2002b).

(5) $$\begin{array}{rcl} log\frac{k_{N}}{k_{N}^{0\;}} & = & \;\beta_{2}\;\left(pK_{a}-\;pK_{a}^{0}\right)-log\;log\;\left[\frac{\left(1\;+a\right)}{2}\right];\\ log\;log\;a\; & = & \left(\beta_{2}-\;\beta_{1}\right)\left(pK_{a}-\;pK_{a}^{0}\right) \end{array}$$

The Brönsted slopes are β_1_ and β_2_ at high and low *pK*_*a*_, respectively and $k_{N}^{0}$ and $pK_{a}^{0}$ are the corresponding values at the center of curvature. The Brönsted curve for SAA shown a $pK_{a}^{0}$ value of 9.0 and 9.15 for PA, respectively. Note that, the *pK*_*a*_ values for the complete series of nucleophiles of this study, cover a range of *pK*_*a*_ values in aqueous media between 5.81 to 11.24 (SAA) and 5.70 to 10.66 (PA), respectively. The higher${\;pK}_{a}^{0}$ values obtained could be explained by the nature of the PG. The presence of a strong electron withdrawing group (-NO_2_) and its high electronic delocalization would promote the high reactivity of the reacting pair allowing the LG departure and the nucleophilic attack at the same time promoting a *c*S_N_Ar route for these reactions.

Finally, the nature of the reacting pair is determinant on the reaction channel and it may be used to predict the degree of polar character at the TS structure. The latter, is achieved on the basis of the electronic information given by the nature of the LG and PG, solvent effects, strength of the nucleophile, electrofugality and nucleofugality of the fragment associated to the reaction. The studied reactions have: basic amines, a good LG (chlorine atom) and heterocyclic ring. The high nucleophilicity of these amines added to the high nucleofugality of the chlorine atom promoted by a heterocyclic ring highly stabilized by nitrogen atoms that improve the electrophilicity of the ipso carbon in the PG suggests that the intermediate specie is not stable and the reactions proceed without a MC intermediate only with one TS structure suggesting a concerted route for the studied reactions.

In summary, the subtlety of the mathematical treatment of the kinetic data and the analysis of the reacting pair suggest a concerted mechanism for the studied reactions of both amine series. A pertinent alternative is to complete the experimental study with reliable theoretical and computational studies in order to validate the proposed reaction route, specifically oriented in these amines that produce the curvature on the Brönsted type-plots. However, a complete and detailed experimental work is enough to validate a mechanistic study.

## Conclusions

The mechanism of the S_N_Ar reaction between PA and SAA with 2-chloro-5-nitro pyrimidine, respectively have been elucidated by kinetic measurements. The first approach of the kinetic data reveals a non-catalyzed pathway. Then, the Brönsted type-plots analysis opened a complete discussion based on the subtlety of the mathematical treatment of the kinetic data suggesting a concerted mechanism for both amines discarding a nucleophilic attack as RDS on the reaction mechanism. The information given by curved Brönsted type-plots was complemented with the analysis of the chemical structures of the reacting pair and its relationships with the reaction pathway, validating the proposed concerted pathway. This article emphasizes the importance of Brönsted type-plots analysis highlighting the significance of the β value as a measure to determine the bond formation and the reaction mechanisms.

## Data Availability Statement

All datasets generated for this study are included in the article/Supplementary Material.

## Author Contributions

BO performed kinetic experiments, RT synthesized the reaction products and PC designed the experiments, analyzed the results, wrote and revised the manuscript. All the authors have approved the final revised manuscript. PC on behalf of The Collaborative Working Group.

## Conflict of Interest

The authors declare that the research was conducted in the absence of any commercial or financial relationships that could be construed as a potential conflict of interest.
