# Supplementary material for: Experimental Analyses Emphasize the Stability of the Meisenheimer Complex in a SNAr Reaction Toward Trends in Reaction Pathways
Source: Front Chem. 2020 Jul 10;8:583. doi: 10.3389/fchem.2020.00583 (PMC7366874; doi:10.3389/fchem.2020.00583)

Supplementary Material

**Experimental Analyses Emphasize the Stability of the Meisenheimer Complex in a S_N_Ar Reaction towards trends in Reaction Pathways.**

**Paola R. Campodónico*^1^, Belén Olivares^1^ and Ricardo A. Tapia^2^**

^1^Centro de Química Médica. Facultad de Medicina. Clínica Alemana Universidad del Desarrollo. Código Postal 7710162. Santiago. Chile. ^2^Facultad de Química y Farmacia. Pontificia Universidad Católica de Chile. Casilla 306, Santiago 6094411. Chile.

[pcampodonico@udd.cl](mailto:migazitu@uc.cl)

**Table of Contents**

| Figure S1 | Plot of *k*_obs_ against free amine concentration [RNH_2_]_F_ for the reaction of 2-chloro-5-nitro pyrimidine with propylamine at three pH values in aqueous solution at 25.0 °C and ionic strength 0.2M (KCl). | S7 |
| --- | --- | --- |
| Figure S2 | Plot of *k*_obs_ against free amine concentration [RNH_2_]_F_ for the reaction of 2-chloro-5-nitro pyrimidine with glycine at three pH values in aqueous solution at 25.0 °C and ionic strength 0.2M (KCl). | S7 |
| Figure S3 | Plot of *k*_obs_ against free amine concentration [RNH_2_]_F_ for the reaction of 2-chloro-5-nitro pyrimidine with ethanolamine at three pH values in aqueous solution at 25.0 °C and ionic strength 0.2M (KCl). | S7 |
| Figure S4 | Plot of *k*_obs_ against free amine concentration [RNH_2_]_F_ for the reaction of 2-chloro-5-nitro pyrimidine with benzylamine at three pH values in aqueous solution at 25.0 °C and ionic strength 0.2M (KCl). | S8 |
| Figure S5 | Plot of *k*_obs_ against free amine concentration [RNH_2_]_F_ for the reaction of 2-chloro-5-nitro pyrimidine with glycine ethyl ester at three pH values in aqueous solution at 25.0 °C and ionic strength 0.2M (KCl). | S8 |
| Figure S6 | Plot of *k*_obs_ against free amine concentration [RNH_2_]_F_ for the reaction of 2-chloro-5-nitro pyrimidine with 1.2-diamino propane at three pH values in aqueous solution at 25.0 °C and ionic strength 0.2M (KCl). | S8 |
| Figure S7 | Plot of *k*_obs_ against free amine concentration [RNH_2_]_F_ for the reaction of 2-chloro-5-nitro pyrimidine with trifluoroethylamine at three pH values in aqueous solution at 25.0 °C and ionic strength 0.2M (KCl). | S9 |
| Figure S8 | Plot of *k*_obs_ against free amine concentration [R_2_NH]_F_ for the reaction of 2-chloro-5-nitro pyrimidine with piperidine at three pH values in aqueous solution at 25.0 °C and ionic strength 0.2M (KCl). | S9 |
| Figure S9 | Plot of *k*_obs_ against free amine concentration [R_2_NH]_F_ for the reaction of 2-chloro-5-nitro pyrimidine with piperazine at three pH values in aqueous solution at 25.0 °C and ionic strength 0.2M (KCl). | S9 |
| Figure S10 | Plot of *k*_obs_ against free amine concentration [R_2_NH]_F_ for the reaction of 2-chloro-5-nitro pyrimidine with 1-(-2-hidroxyethyl) piperazine at three pH values in aqueous solution at 25.0 °C and ionic strength 0.2M (KCl). | S10 |
| Figure S11 | Plot of *k*_obs_ against free amine concentration [R_2_NH]_F_ for the reaction of 2-chloro-5-nitro pyrimidine with morpholine at three pH values in aqueous solution at 25.0 °C and ionic strength 0.2M (KCl). | S10 |
| Figure S12 | Plot of *k*_obs_ against free amine concentration [R_2_NH]_F_ for the reaction of 2-chloro-5-nitro pyrimidine with 1-formylpiperazine at three pH values in aqueous solution at 25.0 °C and ionic strength 0.2M (KCl). | S10 |
| Figure S13 | Plot of *k*_obs_ against free amine concentration [R_2_NH]_F_ for the reaction of 2-chloro-5-nitro pyrimidine with piperazinium ion at three pH values in aqueous solution at 25.0 °C and ionic strength 0.2M (KCl). | S11 |
| Figure S14. | Plot of *k*_obs_ against free amine concentration [R_2_NH]_F_ for the reaction of 2-chloro-5-nitro pyrimidine with piperidine at three pH values in aqueous solution at 25.0 °C and ionic strength 0.2M (KCl). Kinetic data were performed in stop flow equipment. | S11 |
| Table S1 | Kinetic data for the reaction of 2-chloro-5-nitro pyrimidine with propylamine in aqueous solution at 25°C±0.1°C and pH=10.59 | S12 |
| Table S2 | Kinetic data for the reaction of 2-chloro-5-nitro pyrimidine with propylamine in aqueous solution at 25°C±0.1°C and pH=10.89. | S12 |
| Table S3 | Kinetic data for the reaction of 2-chloro-5-nitro pyrimidine with propylamine in aqueous solution at 25°C±0.1°C and pH=11.19. | S13 |
| Table S4 | Kinetic data for the reaction of 2-chloro-5-nitro pyrimidine with glycine in aqueous solution at 25°C±0.1°C and pH=9.46. | S13 |
| Table S5 | Kinetic data for the reaction of 2-chloro-5-nitro pyrimidine with glycine in aqueous solution at 25°C±0.1°C and pH=10.06. | S14 |
| Table S6 | Kinetic data for the reaction of 2-chloro-5-nitro pyrimidine with glycine in aqueous solution at 25°C±0.1°C and pH=10.36. | S14 |
| Table S7 | Kinetic data for the reaction of 2-chloro-5-nitro pyrimidine with ethanolamine in aqueous solution at 25°C±0.1°C and pH=9.37. | S15 |
| Table S8 | Kinetic data for the reaction of 2-chloro-5-nitro pyrimidine with ethanolamine in aqueous solution at 25°C±0.1°C and pH=9.67. | S15 |
| Table S9 | Kinetic data for the reaction of 2-chloro-5-nitro pyrimidine with ethanolamine in aqueous solution at 25°C±0.1°C and pH=9.97. | S16 |
| Table S10 | Kinetic data for the reaction of 2-chloro-5-nitro pyrimidine with benzylamine in aqueous solution at 25°C±0.1°C and pH=9.16. | S16 |
| Table S11 | Kinetic data for the reaction of 2-chloro-5-nitro pyrimidine with benzylamine in aqueous solution at 25°C±0.1°C and pH=9.46. | S17 |
| Table S12 | Kinetic data for the reaction of 2-chloro-5-nitro pyrimidine with benzylamine in aqueous solution at 25°C±0.1°C and pH=9.76. | S17 |
| Table S13 | Kinetic data for the reaction of 2-chloro-5-nitro pyrimidine with glycine ethyl ester in aqueous solution at 25°C±0.1°C and pH=7.38. | S18 |
| Table S14 | Kinetic data for the reaction of 2-chloro-5-nitro pyrimidine with glycine ethyl ester in aqueous solution at 25°C±0.1°C and pH=7.68. | S18 |
| Table S15 | Kinetic data for the reaction of 2-chloro-5-nitro pyrimidine with glycine ethyl ester in aqueous solution at 25°C±0.1°C and pH=7.98. | S19 |
| Table S16 | Kinetic data for the reaction of 2-chloro-5-nitro pyrimidine with 1.2-diamino propane in aqueous solution at 25°C±0.1°C and pH=6.83 | S19 |
| Table S17 | Kinetic data for the reaction of 2-chloro-5-nitro pyrimidine with 1.2-diamino propane in aqueous solution at 25°C±0.1°C and pH=7.13 | S20 |
| Table S18 | Kinetic data for the reaction of 2-chloro-5-nitro pyrimidine with 1.2-diamino propane in aqueous solution at 25°C±0.1°C and pH=7.43 | S20 |
| Table S19 | Kinetic data for the reaction of 2-chloro-5-nitro pyrimidine with trifluoroethylamine in aqueous solution at 25°C±0.1°C and pH=5.4. | S21 |
| Table S20 | Kinetic data for the reaction of 2-chloro-5-nitro pyrimidine with trifluoroethylamine in aqueous solution at 25°C±0.1°C and pH=5.7. | S21 |
| Table S21 | Kinetic data for the reaction of 2-chloro-5-nitro pyrimidine with trifluoroethylamine in aqueous solution at 25°C±0.1°C and pH=6.0. | S22 |
| Table S22 | Kinetic data for the reaction of 2-chloro-5-nitro pyrimidine with piperidine in aqueous solution at 25°C±0.1°C and pH=10.94. | S22 |
| Table S23 | Kinetic data for the reaction of 2-chloro-5-nitro pyrimidine with piperidine in aqueous solution at 25°C±0.1°C and pH=11.24. | S23 |
| Table S24 | Kinetic data for the reaction of 2-chloro-5-nitro pyrimidine with piperidine in aqueous solution at 25°C±0.1°C and pH=11.54. | S23 |
| Table S25 | Kinetic data for the reaction of 2-chloro-5-nitro pyrimidine with piperazine in aqueous solution at 25°C±0.1°C and pH=9.64. | S24 |
| Table S26 | Kinetic data for the reaction of 2-chloro-5-nitro pyrimidine with piperazine in aqueous solution at 25°C±0.1°C and pH=9.64. | S24 |
| Table S27 | Kinetic data for the reaction of 2-chloro-5-nitro pyrimidine with piperazine in aqueous solution at 25°C±0.1°C and pH=9.64. | S25 |
| Table S28 | Kinetic data for the reaction of 2-chloro-5-nitro pyrimidine with 1-(2-hidroxyethyl)piperazine in aqueous solution at 25°C±0.1°C and pH=9.08. | S25 |
| Table S29 | Kinetic data for the reaction of 2-chloro-5-nitro pyrimidine with 1-(2-hidroxyethyl)piperazine in aqueous solution at 25°C±0.1°C and pH=9.38. | S26 |
| Table S30 | Kinetic data for the reaction of 2-chloro-5-nitro pyrimidine with 1-(2-hidroxyethyl)piperazine in aqueous solution at 25°C±0.1°C and pH=9.68. | S27 |
| Table S31 | Kinetic data for the reaction of 2-chloro-5-nitro pyrimidine with morpholine in aqueous solution at 25°C±0.1°C and pH=8.48. | S27 |
| Table S32 | Kinetic data for the reaction of 2-chloro-5-nitro pyrimidine with morpholine in aqueous solution at 25°C±0.1°C and pH=8.78. | S28 |
| Table S33 | Kinetic data for the reaction of 2-chloro-5-nitro pyrimidine with morpholine in aqueous solution at 25°C±0.1°C and pH=9.08. | S28 |
| Table S34 | Kinetic data for the reaction of 2-chloro-5-nitro pyrimidine with 1-formylpiperazine in aqueous solution at 25°C±0.1°C and pH=7.4. | S29 |
| Table S35 | Kinetic data for the reaction of 2-chloro-5-nitro pyrimidine with 1-formylpiperazine in aqueous solution at 25°C±0.1°C and pH=7.7. | S29 |
| Table S36 | Kinetic data for the reaction of 2-chloro-5-nitro pyrimidine with 1-formylpiperazine in aqueous solution at 25°C±0.1°C and pH=7.9. | S30 |
| Table S37 | Kinetic data for the reaction of 2-chloro-5-nitro pyrimidine with piperazinium ion in aqueous solution at 25°C±0.1°C and pH=5.51. | S30 |
| Table S38 | Kinetic data for the reaction of 2-chloro-5-nitro pyrimidine with piperazinium ion in aqueous solution at 25°C±0.1°C and pH=5.81. | S31 |
| Table S39 | Kinetic data for the reaction of 2-chloro-5-nitro pyrimidine with piperazinium ion in aqueous solution at 25°C±0.1°C and pH=6.11. | S31 |
| Table S40 | Kinetic data for the reaction of 2-chloro-5-nitro pyrimidine with piperidine in aqueous solution at 25°C±0.1°C and pH=10.94 measured in stop flow equipment. | S32 |
| Table S41 | Kinetic data for the reaction of 2-chloro-5-nitro pyrimidine with piperidine in aqueous solution at 25°C±0.1°C and pH=11.24 measured in stop flow equipment. | S32 |
| Table S42 | Kinetic data for the reaction of 2-chloro-5-nitro pyrimidine with piperidine in aqueous solution at 25°C±0.1°C and pH=11.54 measured in stop flow equipment. | S33 |
| Figure S15. | Screen view for the reaction of 2-chloro-5-nitro pyrimidine with 1-(2-hidroxyethyl)piperazine in aqueous solution at 25°C±0.1°C and pH=9.08. | S34 |
| Figure S16. | All the spectra for the reaction of 2-chloro-5-nitro pyrimidine with benzylamine in aqueous solution at 25°C±0.1°C and pH=9.16. | S34 |

All the reactants were commercially available, the reactions were executed at constant temperature and the experiments were carried out several times. Each reaction considered at least 30 kinetic measures in order to determine the nucleophilic rate coefficient (*k_N_*) at 3 pH values, and to discard some catalysis processes given by the reaction media.

Figure S1. Plot of *k*_obs_ against free amine concentration [RNH_2_]_F_ for the reaction of 2-chloro-5-nitro pyrimidine with propylamine at three pH values in aqueous solution at 25.0 °C and ionic strength 0.2M (KCl).

Figure S2. Plot of *k*_obs_ against free amine concentration [RNH_2_]_F_ for the reaction of 2-chloro-5-nitro pyrimidine with glycine at three pH values in aqueous solution at 25.0 °C and ionic strength 0.2M (KCl).

Figure S3. Plot of *k*_obs_ against free amine concentration [RNH_2_]_F_ for the reaction of 2-chloro-5-nitro pyrimidine with ethanolamine at three pH values in aqueous solution at 25.0 °C and ionic strength 0.2M (KCl).

Figure S4. Plot of *k*_obs_ against free amine concentration [RNH_2_]_F_ for the reaction of 2-chloro-5-nitro pyrimidine with benzylamine at three pH values in aqueous solution at 25.0 °C and ionic strength 0.2M (KCl).

Figure S5. Plot of *k*_obs_ against free amine concentration [RNH_2_]_F_ for the reaction of 2-chloro-5-nitro pyrimidine with glycine ethyl ester at three pH values in aqueous solution at 25.0 °C and ionic strength 0.2M (KCl).

Figure S6. Plot of *k*_obs_ against free amine concentration [RNH_2_]_F_ for the reaction of 2-chloro-5-nitro pyrimidine with 1.2-diamino propane at three pH values in aqueous solution at 25.0 °C and ionic strength 0.2M (KCl)

Figure S7. Plot of *k*_obs_ against free amine concentration [RNH_2_]_F_ for the reaction of 2-chloro-5-nitro pyrimidine with trifluoroethylamine at three pH values in aqueous solution at 25.0 °C and ionic strength 0.2M (KCl).

Figure S8. Plot of *k*_obs_ against free amine concentration [R_2_NH]_F_ for the reaction of 2-chloro-5-nitro pyrimidine with piperidine at three pH values in aqueous solution at 25.0 °C and ionic strength 0.2M (KCl).

Figure S9. Plot of *k*_obs_ against free amine concentration [R_2_NH]_F_ for the reaction of 2-chloro-5-nitro pyrimidine with piperazine at three pH values in aqueous solution at 25.0 °C and ionic strength 0.2M (KCl).

Figure S10. Plot of *k*_obs_ against free amine concentration [R_2_NH]_F_ for the reaction of 2-chloro-5-nitro pyrimidine with 1-(-2-hidroxyethyl) piperazine at three pH values in aqueous solution at 25.0 °C and ionic strength 0.2M (KCl).

Figure S11. Plot of *k*_obs_ against free amine concentration [R_2_NH]_F_ for the reaction of 2-chloro-5-nitro pyrimidine with morpholine at three pH values in aqueous solution at 25.0 °C and ionic strength 0.2M (KCl).

Figure S12. Plot of *k*_obs_ against free amine concentration [R_2_NH]_F_ for the reaction of 2-chloro-5-nitro pyrimidine with 1-formylpiperazine at three pH values in aqueous solution at 25.0 °C and ionic strength 0.2M (KCl).

Figure S13. Plot of *k*_obs_ against free amine concentration [R_2_NH]_F_ for the reaction of 2-chloro-5-nitro pyrimidine with piperazinium ion at three pH values in aqueous solution at 25.0 °C and ionic strength 0.2M (KCl).

Figure S14. Plot of *k*_obs_ against free amine concentration [R_2_NH]_F_ for the reaction of 2-chloro-5-nitro pyrimidine with piperidine at three pH values in aqueous solution at 25.0 °C and ionic strength 0.2M (KCl). Kinetic data were performed in stop flow equipment.

Table S1. Kinetic data for the reaction of 2-chloro-5-nitro pyrimidine with propylamine in aqueous solution at 25°C±0.1°C and pH=10.59.

|  | 10^3^ [RNH_2_]_F_ | 10^2^ *k*_obs_ / s^-1^ |
| --- | --- | --- |
| 1 | 1.04 | 1.85 |
| 2 | 2.60 | 3.23 |
| 3 | 4.16 | 4.34 |
| 4 | 57.2 | 5.38 |
| 5 | 72.8 | 6.38 |
| 6 | 88.4 | 7.49 |
| 7 | 104 | 8.56 |

Table S2. Kinetic data for the reaction of 2-chloro-5-nitro pyrimidine with propylamine in aqueous solution at 25°C±0.1°C and pH=10.89.

|  | 10^3^ [RNH_2_]_F_ | 10^2^ *k*_obs_ / s^-1^ |
| --- | --- | --- |
| 1 | 2.71 | 3.60 |
| 2 | 6.79 | 6.80 |
| 3 | 10.9 | 8.74 |
| 4 | 14.9 | 11.1 |
| 5 | 19.0 | 12.0 |
| 6 | 27.1 | 17.0 |

Table S3. Kinetic data for the reaction of 2-chloro-5-nitro pyrimidine with propylamine in aqueous solution at 25°C±0.1°C and pH=11.19.

|  | 10^2^ [RNH_2_]_F_ | 10^3^ *k*_obs_ / s^-1^ |
| --- | --- | --- |
| 1 | 7.08 | 8.78 |
| 2 | 9.74 | 9.71 |
| 3 | 12.4 | 11.3 |
| 4 | 15.1 | 11.5 |
| 5 | 17.7 | 12.7 |

Table S4. Kinetic data for the reaction of 2-chloro-5-nitro pyrimidine with glycine in aqueous solution at 25°C±0.1°C and pH=9.46.

|  | 10^3^ [RNH_2_]_F_ | 10^2^ *k*_obs_ / s^-1^ |
| --- | --- | --- |
| 1 | 2.27 | 1.34 |
| 2 | 5.67 | 2.58 |
| 3 | 9.07 | 4.18 |
| 4 | 12.5 | 5.71 |
| 5 | 15.9 | 6.43 |

Table S5. Kinetic data for the reaction of 2-chloro-5-nitro pyrimidine with glycine in aqueous solution at 25°C±0.1°C and pH=10.06.

|  | 10^3^ [RNH_2_]_F_ | 10^2^ *k*_obs_ / s^-1^ |
| --- | --- | --- |
| 1 | 4.80 | 1.50 |
| 2 | 12.0 | 4.28 |
| 3 | 19.2 | 6.11 |
| 4 | 26.4 | 8.59 |
| 5 | 33.0 | 11.6 |
| 6 | 40.8 | 13.4 |
| 7 | 48.0 | 16.1 |

Table S6. Kinetic data for the reaction of 2-chloro-5-nitro pyrimidine with glycine in aqueous solution at 25°C±0.1°C and pH=10.36.

|  | 10^3^ [RNH_2_]_F_ | 10^2^ *k*_obs_ / s^-1^ |
| --- | --- | --- |
| 1 | 4.37 | 1.79 |
| 2 | 10.9 | 4.26 |
| 3 | 17.5 | 6.87 |
| 4 | 24.0 | 7.68 |
| 5 | 30.6 | 10.5 |
| 6 | 37.2 | 12.3 |

Table S7. Kinetic data for the reaction of 2-chloro-5-nitro pyrimidine with ethanolamine in aqueous solution at 25°C±0.1°C and pH=9.37.

|  | 10^3^ [RNH_2_]_F_ | 10^2^ *k*_obs_ / s^-1^ |
| --- | --- | --- |
| 1 | 2.35 | 0.473 |
| 2 | 5.89 | 0.915 |
| 3 | 9.42 | 1.66 |
| 4 | 13.0 | 2.35 |
| 5 | 16.5 | 3.00 |
| 6 | 20.0 | 3.55 |

Table S8. Kinetic data for the reaction of 2-chloro-5-nitro pyrimidine with ethanolamine in aqueous solution at 25°C±0.1°C and pH=9.67.

|  | 10^3^ [RNH_2_]_F_ | 10^2^ *k*_obs_ / s^-1^ |
| --- | --- | --- |
| 1 | 3.24 | 0.787 |
| 2 | 8.10 | 1.57 |
| 3 | 13.0 | 2.53 |
| 4 | 17.8 | 3.51 |
| 5 | 22.7 | 4.20 |
| 6 | 27.5 | 5.30 |
| 7 | 32.4 | 6.26 |

Table S9. Kinetic data for the reaction of 2-chloro-5-nitro pyrimidine with ethanolamine in aqueous solution at 25°C±0.1°C and pH=9.97.

|  | 10^3^ [RNH_2_]_F_ | 10^2^ *k*_obs_ / s^-1^ |
| --- | --- | --- |
| 1 | 3.95 | 0.799 |
| 2 | 9.86 | 2.32 |
| 3 | 15.8 | 2.92 |
| 4 | 21.7 | 3.90 |
| 5 | 27.6 | 5.20 |
| 6 | 33.5 | 6.40 |
| 7 | 39.5 | 8.13 |

Table S10. Kinetic data for the reaction of 2-chloro-5-nitro pyrimidine with benzylamine in aqueous solution at 25°C±0.1°C and pH=9.16.

|  | 10^3^ [RNH_2_]_F_ | 10^2^ *k*_obs_ / s^-1^ |
| --- | --- | --- |
| 1 | 3.57 | 0.706 |
| 2 | 8.92 | 1.47 |
| 3 | 14.3 | 3.32 |
| 4 | 19.6 | 4.64 |
| 5 | 25.0 | 6.02 |
| 6 | 30.3 | 7.56 |
| 7 | 35.7 | 9.07 |

Table S11. Kinetic data for the reaction of 2-chloro-5-nitro pyrimidine with benzylamine in aqueous solution at 25°C±0.1°C and pH=9.46.

|  | 10^3^ [RNH_2_]_F_ | 10^2^ *k*_obs_ / s^-1^ |
| --- | --- | --- |
| 1 | 4.17 | 0.921 |
| 2 | 10.4 | 2.29 |
| 3 | 16.7 | 4.35 |
| 4 | 23.0 | 5.88 |
| 5 | 29.2 | 7.62 |
| 6 | 35.5 | 9.24 |
| 7 | 41.7 | 10.6 |

Table S12. Kinetic data for the reaction of 2-chloro-5-nitro pyrimidine with benzylamine in aqueous solution at 25°C±0.1°C and pH=9.76.

|  | 10^3^ [RNH_2_]_F_ | 10^2^ *k*_obs_ / s^-1^ |
| --- | --- | --- |
| 1 | 5.32 | 1.32 |
| 2 | 13.3 | 3.17 |
| 3 | 21.3 | 5.68 |
| 4 | 29.3 | 8.01 |
| 5 | 37.2 | 10.3 |
| 6 | 45.2 | 12.8 |

Table S13. Kinetic data for the reaction of 2-chloro-5-nitro pyrimidine with glycine ethyl ester in aqueous solution at 25°C±0.1°C and pH=7.38.

|  | 10^3^ [RNH_2_]_F_ | 10^2^ *k*_obs_ / s^-1^ |
| --- | --- | --- |
| 1 | 1.29 | 0.0661 |
| 2 | 3.23 | 0.170 |
| 3 | 5.16 | 0.269 |
| 4 | 7.10 | 0.341 |

Table S14. Kinetic data for the reaction of 2-chloro-5-nitro pyrimidine with glycine ethyl ester in aqueous solution at 25°C±0.1°C and pH=7.68.

|  | 10^3^ [RNH_2_]_F_ | 10^2^ *k*_obs_ / s^-1^ |
| --- | --- | --- |
| 1 | 2.12 | 0.091 |
| 2 | 5.30 | 0.229 |
| 3 | 8.48 | 0.377 |
| 4 | 11.7 | 0.495 |
| 5 | 14.8 | 0.619 |
| 6 | 18.0 | 0.722 |
| 7 | 21.2 | 0.832 |

Table S15. Kinetic data for the reaction of 2-chloro-5-nitro pyrimidine with glycine ethyl ester in aqueous solution at 25°C±0.1°C and pH=7.98.

|  | 10^3^ [RNH_2_]_F_ | 10^2^ *k*_obs_ / s^-1^ |
| --- | --- | --- |
| 1 | 2.87 | 0.132 |
| 2 | 7.17 | 0.313 |
| 3 | 11.5 | 0.464 |
| 4 | 15.8 | 0.708 |
| 5 | 20.1 | 0.922 |
| 6 | 24.4 | 1.07 |
| 7 | 28.7 | 1.31 |

Table S16. Kinetic data for the reaction of 2-chloro-5-nitro pyrimidine with 1.2-diamino propane in aqueous solution at 25°C±0.1°C and pH=6.83.

|  | 10^3^ [RNH_2_]_F_ | 10^2^ *k*_obs_ / s^-1^ |
| --- | --- | --- |
| 1 | 5.96 | 0.129 |
| 2 | 14.9 | 0.324 |
| 3 | 23.8 | 0.539 |
| 4 | 32.8 | 0.796 |
| 5 | 41.7 | 1.01 |
| 6 | 50.7 | 1.23 |
| 7 | 59.6 | 1.49 |

Table S17. Kinetic data for the reaction of 2-chloro-5-nitro pyrimidine with 1.2-diamino propane in aqueous solution at 25°C±0.1°C and pH=7.13.

|  | 10^3^ [RNH_2_]_F_ | 10^2^ *k*_obs_ / s^-1^ |
| --- | --- | --- |
| 1 | 8.98 | 0.166 |
| 2 | 22.5 | 0.427 |
| 3 | 35.9 | 0.678 |
| 4 | 49.4 | 1.05 |
| 5 | 62.9 | 1.50 |
| 6 | 76.4 | 1.68 |
| 7 | 89.8 | 2.04 |

Table S18. Kinetic data for the reaction of 2-chloro-5-nitro pyrimidine with 1.2-diamino propane in aqueous solution at 25°C±0.1°C and pH=7.43.

|  | 10^3^ [RNH_2_]_F_ | 10^2^ *k*_obs_ / s^-1^ |
| --- | --- | --- |
| 1 | 11.5 | 0.212 |
| 2 | 16.5 | 0.264 |
| 3 | 41.1 | 0.671 |
| 4 | 63.0 | 1.29 |
| 5 | 80.2 | 1.71 |
| 6 | 97.4 | 2.06 |
| 7 | 115 | 2.53 |

Table S19. Kinetic data for the reaction of 2-chloro-5-nitro pyrimidine with trifluoroethylamine in aqueous solution at 25°C±0.1°C and pH=5.4.

|  | 10^3^ [RNH_2_]_F_ | 10^2^ *k*_obs_ / s^-1^ |
| --- | --- | --- |
| 1 | 3.30 | 0.0107 |
| 2 | 8.25 | 0.0253 |
| 3 | 13.2 | 0.0400 |
| 4 | 18.2 | 0.0520 |
| 5 | 23.1 | 0.0685 |
| 6 | 28.1 | 0.0789 |
| 7 | 33.0 | 0.0973 |

Table S20. Kinetic data for the reaction of 2-chloro-5-nitro pyrimidine with trifluoroethylamine in aqueous solution at 25°C±0.1°C and pH=5.7.

|  | 10^3^ [RNH_2_]_F_ | 10^2^ *k*_obs_ / s^-1^ |
| --- | --- | --- |
| 1 | 5.00 | 0.0142 |
| 2 | 12.5 | 0.0356 |
| 3 | 20.0 | 0.0514 |
| 4 | 27.5 | 0.0700 |
| 5 | 35.0 | 0.0978 |
| 6 | 42.5 | 0.114 |
| 7 | 50.0 | 0.139 |

Table S21. Kinetic data for the reaction of 2-chloro-5-nitro pyrimidine with trifluoroethylamine in aqueous solution at 25°C±0.1°C and pH=6.0.

|  | 10^3^ [RNH_2_]_F_ | 10^2^ *k*_obs_ / s^-1^ |
| --- | --- | --- |
| 1 | 5.87 | 0.0149 |
| 2 | 14.7 | 0.0339 |
| 3 | 23.5 | 0.0520 |
| 4 | 32.3 | 0.0730 |
| 5 | 41.1 | 0.0929 |
| 6 | 49.9 | 0.110 |
| 7 | 58.7 | 0.132 |

Table S22. Kinetic data for the reaction of 2-chloro-5-nitro pyrimidine with piperidine in aqueous solution at 25°C±0.1°C and pH=10.94.

|  | 10^3^ [R_2_NH]_F_ | 10^2^ *k*_obs_ / s^-1^ |
| --- | --- | --- |
| 1 | 0.196 | 8.50 |
| 2 | 0.490 | 12.0 |
| 3 | 0.784 | 14.6 / 15.7 |
| 4 | 1.08 | 21.2 / 20.2 |
| 5 | 1.37 | 21.5 |
| 6 | 1.67 | 22.8 / 24.1 |
| 7 | 1.96 | 30.6 |

Table S23. Kinetic data for the reaction of 2-chloro-5-nitro pyrimidine with piperidine in aqueous solution at 25°C±0.1°C and pH=11.24.

|  | 10^3^ [R_2_NH]_F_ | 10^2^ *k*_obs_ / s^-1^ |
| --- | --- | --- |
| 1 | 0.433 | 14.4 / 12.3 |
| 2 | 1.08 | 20.9 / 17.8 |
| 3 | 1.73 | 25.4 / 25.1 |
| 4 | 2.38 | 33.6 / 32.2 |
| 5 | 3.68 | 44.8 / 41.2 |
| 6 | 4.33 | 53.6 |

Table S24. Kinetic data for the reaction of 2-chloro-5-nitro pyrimidine with piperidine in aqueous solution at 25°C±0.1°C and pH=11.54.

|  | 10^3^ [R_2_NH]_F_ | 10^2^ *k*_obs_ / s^-1^ |
| --- | --- | --- |
| 1 | 2.42 | 34.2 |
| 2 | 3.08 | 39.3 |
| 3 | 2.42 | 32.5 |
| 4 | 3.75 | 46.1 |

Table S25. Kinetic data for the reaction of 2-chloro-5-nitro pyrimidine with piperazine in aqueous solution at 25°C±0.1°C and pH=9.64.

|  | 10^3^ [R_2_NH]_F_ | 10^2^ *k*_obs_ / s^-1^ |
| --- | --- | --- |
| 1 | 0.201 | 2.94 |
| 2 | 0.502 | 8.48 |
| 3 | 0.803 | 14.4 |
| 4 | 1.10 | 16.8 /17.2 |
| 5 | 1.41 | 21.5 / 21.6 |
| 6 | 1.71 | 24.9 / 23.8 |
| 7 | 2.01 | 30.8 |

Table S26. Kinetic data for the reaction of 2-chloro-5-nitro pyrimidine with piperazine in aqueous solution at 25°C±0.1°C and pH=9.94.

|  | 10^3^ [R_2_NH]_F_ | 10^2^ *k*_obs_ / s^-1^ |
| --- | --- | --- |
| 1 | 0.302 | 4.17 |
| 2 | 0.755 | 9.83 |
| 3 | 1.21 | 14.3 / 15.8 |
| 4 | 1.66 | 19.7 / 21.9 |
| 5 | 2.11 | 33.2 / 25.8 |
| 6 | 2.57 | 29.0 / 31.6 |
| 7 | 3.02 | 39.4 |

Table S27. Kinetic data for the reaction of 2-chloro-5-nitro pyrimidine with piperazine in aqueous solution at 25°C±0.1°C and pH=10.24.

|  | 10^3^ [R_2_NH]_F_ | 10^2^ *k*_obs_ / s^-1^ |
| --- | --- | --- |
| 1 | 0.428 | 6.58 / 6.00 |
| 2 | 1.07 | 13.9 / 14.3 |
| 3 | 1.71 | 24.9/ 23.1 |
| 4 | 2.35 | 29.7 / 35.2 |
| 5 | 2.99 | 38.5 |
| 6 | 3.64 | 52.5 |

Table S28. Kinetic data for the reaction of 2-chloro-5-nitro pyrimidine with 1-(2-hidroxyethyl)piperazine in aqueous solution at 25°C±0.1°C and pH=9.08.

|  | 10^3^ [R_2_NH]_F_ | 10^2^ *k*_obs_ / s^-1^ |
| --- | --- | --- |
| 1 | 0.306 | 1.68 |
| 2 | 0.765 | 4.59 |
| 3 | 1.22 | 7.65 |
| 4 | 1.68 | 10.4 |
| 5 | 2.14 | 12.0 |
| 6 | 2.60 | 13.2 /12.5 |
| 7 | 3.06 | 15.8 / 14.3 |

Table S29. Kinetic data for the reaction of 2-chloro-5-nitro pyrimidine with 1-(2-hidroxyethyl)piperazine in aqueous solution at 25°C±0.1°C and pH=9.38.

|  | 10^3^ [R_2_NH]_F_ | 10^2^ *k*_obs_ / s^-1^ |
| --- | --- | --- |
| 1 | 0.303 | 1.97 |
| 2 | 0.453 | 2.63 |
| 3 | 0.759 | 5.21 |
| 4 | 1.13 | 6.23 |
| 5 | 1.21 | 8.46 |
| 6 | 1.81 | 9.76 |
| 7 | 2.49 | 12.8 |
| 8 | 3.17 | 17.1 |
| 9 | 3.85 | 21.2 |
| 10 | 4.53 | 24.7 |

Table S30. Kinetic data for the reaction of 2-chloro-5-nitro pyrimidine with 1-(2-hidroxyethyl)piperazine in aqueous solution at 25°C±0.1°C and pH=9.68.

|  | 10^3^ [R_2_NH]_F_ | 10^2^ *k*_obs_ / s^-1^ |
| --- | --- | --- |
| 1 | 0.382 | 2.13 |
| 2 | 0.955 | 4.83 |
| 3 | 1.53 | 7.60 |
| 4 | 2.10 | 12.1 |
| 5 | 2.67 | 13.4 |
| 6 | 3.25 | 15.7 |
| 7 | 3.82 | 18.8 |

Table S31. Kinetic data for the reaction of 2-chloro-5-nitro pyrimidine with morpholine in aqueous solution at 25°C±0.1°C and pH=8.48.

|  | 10^3^ [R_2_NH]_F_ | 10^2^ *k*_obs_ / s^-1^ |
| --- | --- | --- |
| 1 | 0.186 | 0.915 |
| 2 | 0.464 | 2.48 |
| 3 | 0.742 | 4.35 |
| 4 | 1.02 | 5.04 |
| 5 | 1.30 | 6.41 |
| 6 | 1.58 | 7.27 |
| 7 | 1.86 | 8.90 |

Table S32. Kinetic data for the reaction of 2-chloro-5-nitro pyrimidine with morpholine in aqueous solution at 25°C±0.1°C and pH=8.78.

|  | 10^3^ [R_2_NH]_F_ | 10^2^ *k*_obs_ / s^-1^ |
| --- | --- | --- |
| 1 | 0.303 | 1.34 |
| 2 | 0.758 | 3.03 |
| 3 | 1.21 | 5.15 |
| 4 | 1.67 | 6.70 |
| 5 | 2.12 | 8.65 |
| 6 | 2.58 | 10.6 |
| 7 | 3.03 | 12.4 |

Table S33. Kinetic data for the reaction of 2-chloro-5-nitro pyrimidine with morpholine in aqueous solution at 25°C±0.1°C and pH=9.08.

|  | 10^3^ [R_2_NH]_F_ | 10^2^ *k*_obs_ / s^-1^ |
| --- | --- | --- |
| 1 | 0.157 | 0.609 |
| 2 | 0.313 | 1.11 |
| 3 | 0.939 | 3.35 |
| 4 | 1.17 | 6.71 |
| 5 | 2.92 | 12.6 |
| 6 | 4.67 | 18.9 |
| 7 | 6.43 | 27.7 |

Table S34. Kinetic data for the reaction of 2-chloro-5-nitro pyrimidine with 1-formylpiperazine in aqueous solution at 25°C±0.1°C and pH=7.4.

|  | 10^3^ [R_2_NH]_F_ | 10^2^ *k*_obs_ / s^-1^ |
| --- | --- | --- |
| 1 | 0.472 | 0.980 |
| 2 | 2.36 | 4.60 |
| 3 | 3.78 | 7.74 |
| 4 | 5.20 | 10.0 |
| 5 | 6.61 | 13.5 |
| 6 | 8.03 | 14.8 |
| 7 | 9.45 | 18.9 |

Table S35. Kinetic data for the reaction of 2-chloro-5-nitro pyrimidine with 1-formylpiperazine in aqueous solution at 25°C±0.1°C and pH=7.7.

|  | 10^3^ [R_2_NH]_F_ | 10^2^ *k*_obs_ / s^-1^ |
| --- | --- | --- |
| 1 | 6.26 | 15.3 |
| 2 | 8.60 | 17.9 |
| 3 | 10.9 | 23.0 |
| 4 | 13.3 | 26.5 |
| 5 | 15.6 | 31.3 |

Table S36. Kinetic data for the reaction of 2-chloro-5-nitro pyrimidine with 1-formylpiperazine in aqueous solution at 25°C±0.1°C and pH=7.9.

|  | 10^3^ [R_2_NH]_F_ | 10^2^ *k*_obs_ / s^-1^ |
| --- | --- | --- |
| 1 | 1.88 | 3.60 |
| 2 | 4.69 | 8.15 |
| 3 | 7.51 | 14.0 |
| 4 | 10.3 | 19.5 |
| 5 | 13.1 | 26.0 |
| 6 | 16.0 | 31.8 |

Table S37. Kinetic data for the reaction of 2-chloro-5-nitro pyrimidine with piperazinium ion in aqueous solution at 25°C±0.1°C and pH=5.51.

|  | 10^3^ [R_2_NH]_F_ | 10^2^ *k*_obs_ / s^-1^ |
| --- | --- | --- |
| 1 | 1.08 | 0.222 |
| 2 | 2.69 | 0.514 |
| 3 | 4.30 | 0.815 |
| 4 | 5.92 | 1.22 |
| 5 | 5.87 | 1.18 |

Table S38. Kinetic data for the reaction of 2-chloro-5-nitro pyrimidine with piperazinium ion in aqueous solution at 25°C±0.1°C and pH=5.81.

|  | 10^3^ [R_2_NH]_F_ | 10^2^ *k*_obs_ / s^-1^ |
| --- | --- | --- |
| 1 | 1.74 | 0.444 |
| 2 | 4.34 | 0.726 |
| 3 | 6.94 | 1.17 |
| 4 | 9.55 | 1.42 |
| 5 | 12.1 | 1.96 |
| 6 | 14.8 | 2.92 |
| 7 | 17.4 | 3.41 |

Table S39. Kinetic data for the reaction of 2-chloro-5-nitro pyrimidine with piperazinium ion in aqueous solution at 25°C±0.1°C and pH=6.11.

|  | 10^3^ [R_2_NH]_F_ | 10^2^ *k*_obs_ / s^-1^ |
| --- | --- | --- |
| 1 | 2.75 | 0.392 |
| 2 | 6.88 | 0.906 |
| 3 | 11.0 | 1.89 |
| 4 | 15.1 | 2.68 |
| 5 | 19.3 | 3.52 |
| 6 | 23.4 | 4.28 |
| 7 | 27.5 | 4.94 |

Table S40. Kinetic data for the reaction of 2-chloro-5-nitro pyrimidine with piperidine in aqueous solution at 25°C±0.1°C and pH=10.94 measured in stop flow equipment.

|  | 10^3^ [R_2_NH]_F_ | 10^2^ *k*_obs_ / s^-1^ |
| --- | --- | --- |
| 1 | 0.215 | 5.25 |
| 2 | 0.537 | 8.20 |
| 3 | 0.859 | 10.2 |
| 4 | 1.18 | 11.7 |
| 5 | 1.50 | 12.6 |
| 6 | 1.82 | 14.1 |
| 7 | 2.15 | 16.7 |

Table S41. Kinetic data for the reaction of 2-chloro-5-nitro pyrimidine with piperidine in aqueous solution at 25°C±0.1°C and pH=11.24 measured in stop flow equipment.

|  | 10^3^ [R_2_NH]_F_ | 10^2^ *k*_obs_ / s^-1^ |
| --- | --- | --- |
| 1 | 0.284 | 5.68 |
| 2 | 0.709 | 7.30 |
| 3 | 1.13 | 9.83 |
| 4 | 1.56 | 11.8 |
| 5 | 1.99 | 13.5 |
| 6 | 2.41 | 16.3 |
| 7 | 1.87 | 18.7 |

Table S42. Kinetic data for the reaction of 2-chloro-5-nitro pyrimidine with piperidine in aqueous solution at 25°C±0.1°C and pH=11.54 measured in stop flow equipment.

|  | 10^3^ [R_2_NH]_F_ | 10^2^ *k*_obs_ / s^-1^ |
| --- | --- | --- |
| 1 | 0.40 | 7.79 |
| 2 | 0.99 | 9.70 |
| 3 | 1.16 | 11.6 |
| 4 | 2.20 | 14.8 |
| 5 | 2.80 | 18.7 |
| 6 | 3.40 | 22.0 |
| 7 | 4.00 | 27.5 |

Figure S15. Screen view for the reaction of 2-chloro-5-nitro pyrimidine with 1-(2-hidroxyethyl)piperazine in aqueous solution at 25°C±0.1°C and pH=9.08.


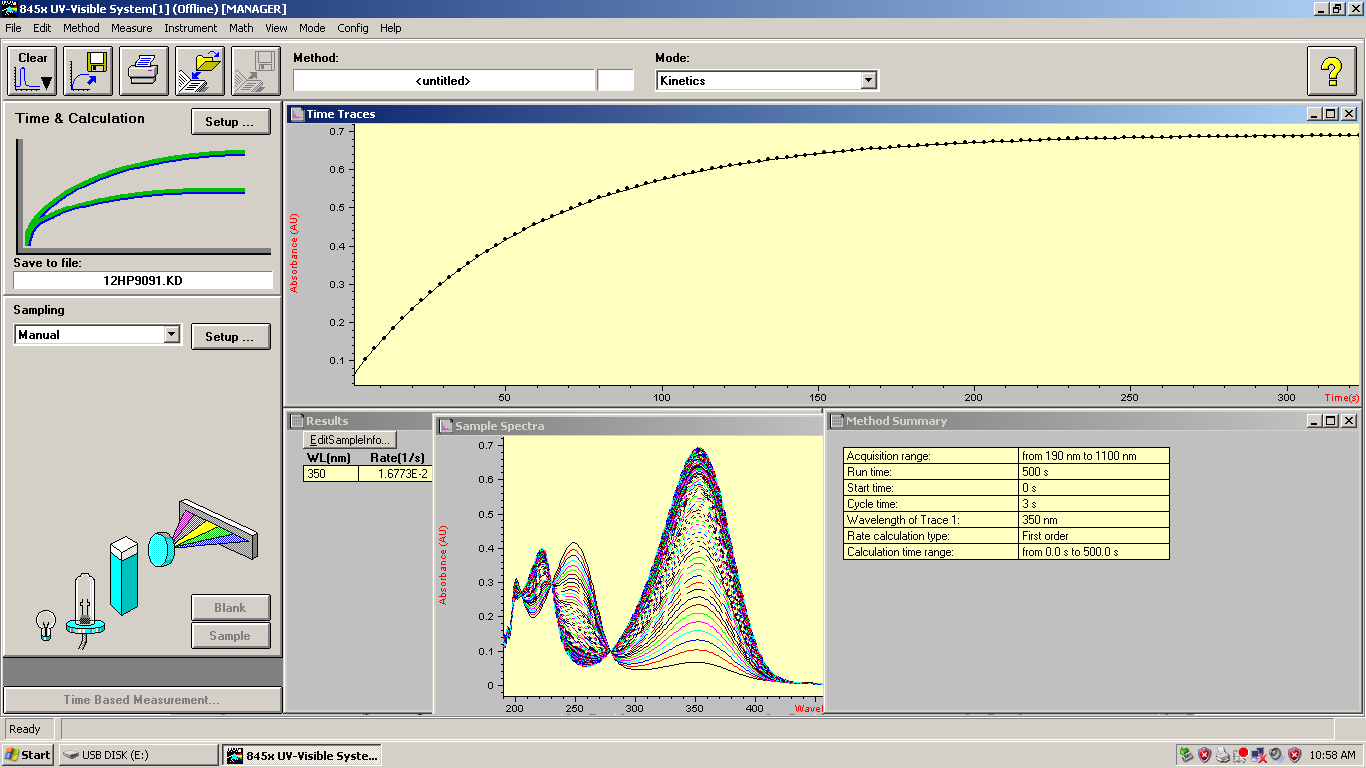


Figure S16. All the spectra for the reaction of 2-chloro-5-nitro pyrimidine with benzylamine in aqueous solution at 25°C±0.1°C and pH=9.16.


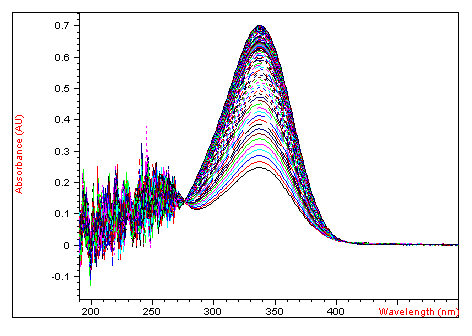

Supplement: Supplementary file 1 [file Table_1.docx]
